# Aiming at the Global Elimination of Viral Hepatitis: Challenges Along the Care Continuum

**DOI:** 10.1093/ofid/ofx252

**Published:** 2017-11-17

**Authors:** Alastair Heffernan, Ella Barber, Nicola A Cook, Asmaa I Gomaa, Yolande X Harley, Christopher R Jones, Aaron G Lim, Zameer Mohamed, Shevanthi Nayagam, Gibril Ndow, Rajiv Shah, Mark W Sonderup, C Wendy Spearman, Imam Waked, Robert J Wilkinson, Simon D Taylor-Robinson

**Affiliations:** 1 Department of Infectious Disease Epidemiology, Faculty of Medicine, Imperial College London, London, UK; 2 Division of Infectious Diseases, Faculty of Medicine, Imperial College London, London, UK; 3 Médecins Sans Frontières, London, UK; 4 Department of Surgery and Cancer, Faculty of Medicine, Imperial College London, London, UK; 5 Hepatology Department, National Liver Institute, Menoufiya University, Shebeen El-Kom, Egypt; 6 Research Office, Faculty of Health Sciences, University of Cape Town, Cape Town, South Africa; 7 Population Health Sciences, Bristol Medical School, University of Bristol, Bristol, UK; 8 Liver and Antiviral Unit, Imperial College Healthcare NHS Trust, St. Mary’s Hospital, London, UK; 9 Hepatitis Unit, Disease Control and Elimination, MRC Unit, Banjul, The Gambia; 10 Infectious Diseases Department, Nottingham University Hospitals NHS Trust, Nottingham, UK; 11 Division of Hepatology, Department of Medicine, Faculty of Health Sciences, University of Cape Town, Cape Town, South Africa; 12 Institute of Infectious Disease and Molecular Medicine, Faculty of Health Sciences, University of Cape Town, Cape Town, South Africa; 13 Tuberculosis Laboratory, The Francis Crick Institute, London, UK

**Keywords:** elimination, hepatitis care continuum; policy, viral hepatitis

## Abstract

A recent international workshop, organized by the authors, analyzed the obstacles facing the ambitious goal of eliminating viral hepatitis globally. We identified several policy areas critical to reaching elimination targets. These include providing hepatitis B birth-dose vaccination to all infants within 24 hours of birth, preventing the transmission of blood-borne viruses through the expansion of national hemovigilance schemes, implementing the lessons learned from the HIV epidemic regarding safe medical practices to eliminate iatrogenic infection, adopting point-of-care testing to improve coverage of diagnosis, and providing free or affordable hepatitis C treatment to all. We introduce Egypt as a case study for rapid testing and treatment scale-up: this country offers valuable insights to policy makers internationally, not only regarding how hepatitis C interventions can be expeditiously scaled-up, but also as a guide for how to tackle the problems encountered with such ambitious testing and treatment programs.

Viral hepatitis was responsible for 1.3 million deaths globally in 2015 and is now the seventh leading cause of mortality, rising from the 10th cause in 1990 [1, 2]. The full burden of disease encompasses not only mortality, but also reduced quality of life for patients (through cirrhosis and associated complications), financial costs of care for individuals and health care systems alike, and economic costs to society as a whole. Despite this burden, viral hepatitis has only in recent years received the attention it merits. Now, with a World Health Organization (WHO) elimination strategy published [1], the international community is at last focused on tackling the twin epidemics of hepatitis B virus (HBV) and hepatitis C virus (HCV).

At a recent conference (the first “Chronic Viral Hepatitis in Africa” conference, Egypt), clinicians and researchers from across the globe discussed the challenges of reaching WHO elimination targets. Considering the hepatitis care continuum (Figure 1) [3, 4], we identified several key areas in which progress must be made before elimination will be reached. We focused on those areas in which improvements are possible (using tools currently available) and on those aspects of treatment and care where the impact of changes in policy or strategy is potentially greatest. This viewpoint is a distillation of these discussions and is written with the aim of informing policy at this critical moment in the formation of national and international viral hepatitis programs.

**Figure 1.**
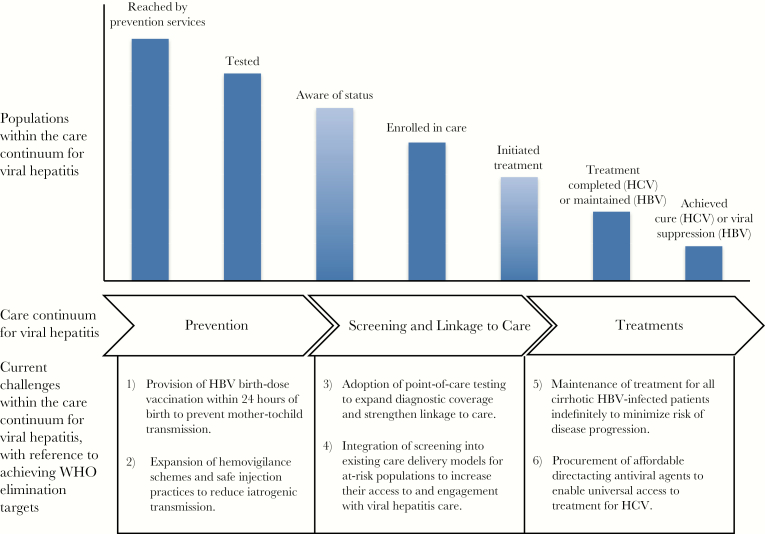
Overview of the WHO care continuum for viral hepatitis and the associated challenges encountered when aiming toward WHO elimination targets, adapted fromn Zhou et al. [3]. Populations within the care continuum for viral hepatitis are as defined within the Global Health Sector Strategy on Viral Hepatitis [4]. Abbreviations: HBV = hepatitis B virus; HCV = hepatitis C virus; WHO = World Health Organization.

## HBV BIRTH-DOSE VACCINATION: CONFRONTING LOST OPPORTUNITIES

Scaling-up of infant vaccination has already had demonstrable impacts on global HBV prevalence [5]. Infant vaccination alone, however, does not prevent mother-to-child transmission (PMTCT). As the risk of chronic hepatitis B (CHB) infection is as high as 90% if infected perinatally [6], effective PMTCT is crucial to reducing incidence.

A key component of a comprehensive PMTCT strategy is birth-dose vaccination. Modeling studies have suggested that an 80% global scale-up of birth-dose vaccination plus infant vaccination, compared with scaling-up of infant vaccination alone, could avert 18.7 million new chronic infections over the next 15 years, highlighting its importance as a PMTCT tool [7]. Monovalent HBV vaccine is inexpensive (US$0.20 per dose), and birth-dose vaccination is likely to be cost-effective [8].

Despite such evidence, global HBV birth-dose vaccine coverage remains low, at 39% [9]. Moreover, vaccines are often administered beyond 24 hours of birth [10], when they are less effective in PMTCT [11, 12]. There are several reasons for this low coverage: the monovalent vaccine is not funded by agencies like GAVI, as the cost of the vaccine falls below their funding threshold [13]; there are significant costs for vaccine delivery [14]; cultural factors may reduce access to health care by women in the postpartum period [15]; and hepatitis B interventions are rarely a public health priority [16], with only 9 countries in sub-Saharan Africa, for example, incorporating birth-dose vaccination into their national policies by 2015 [10, 17].

Innovative approaches exist to improve timely birth-dose vaccination administration, including increasing the number of health staff–attended births [18], ensuring coordination between immunization and maternal health services [19], expanding vaccine management systems [19], using prefilled injectable vaccines for home births [20], promoting awareness of the need for timely HBV vaccination [21], and simplifying the supply chain by, for example, using heat-sensitive labels to allow storage of vaccine outside the cold chain [22, 23].

A substantial scale-up in birth-dose vaccination coverage is pivotal to reaching WHO 2030 elimination targets [1]. This is long overdue, and acceleration of efforts requires a combination of political engagement by governments, financial commitment, and strategic planning to help countries reach these goals.

## IATROGENIC TRANSMISSION: A COST-EFFECTIVE APPROACH TO CURBING TRANSMISSION

Blood and injection safety are fundamental to national viral hepatitis programs [1]. In Africa, for example, the risk of acquiring HCV from blood is 2.5 per 1000 units transfused, compared with 1 per 2–3 million units in high-income countries [24]. In addition, the reuse of injection equipment, inadequate sterilization procedures, lack of universal precautions, sharps injuries, and inadequate medical waste management systems all contribute to the burden of viral hepatitis. To reach WHO targets by 2030, all blood donations should be screened for HIV, HCV, HBV, and syphilis in a quality-assured manner, and 90% of injections should be administered using a safety-engineered device [25].

There are several options for reducing risk of transfusion-transmissible infections (TTIs). Centralized blood transfusion services targeting low-risk, regular, voluntary blood donors should be developed and integrated into health care systems [26]. Although more expensive than replacement donor systems [27], voluntary blood donors represent a safer, more sustainable approach [28] and should contribute at least 80% of all donations to transfusion services [29]. All donations should be screened for TTIs, with external quality assurance, using highly sensitive and specific assays [30, 31]. National hemovigilance systems supported by local transfusion committees enable ongoing surveillance for transfusion-related complications [32], but in low- and middle-income countries (LMICs), only 28% operate hemovigilance systems [33]. Blood safety programs can be cost-effective [34], but cost-effectiveness varies, and this should inform program design: adding antigen-antibody combination tests to reduce the serologically negative window period can be cost-effective [35], whereas TTI predonation screening using rapid diagnostic tests (RDTs) is not considered to be cost-effective [36].

Improving injection safety is also key to reducing iatrogenic transmission: in 2010, approximately 1.7 million new cases of HBV and up to 315 000 new cases of HCV were attributable to unsafe injections [37]. Such infections can be avoided through the use of safety-engineered devices that protect health care workers from hazardous occupational exposures to bodily fluids [38], needle/sharps hygiene and safe disposal, and a ban on needle reuse [39]. Such measures must be delivered alongside education of health care workers in universal precautions and safe waste management systems. National policies for safe and appropriate use of injections are, furthermore, highly cost-effective [40]. Global bodies must take the lead in promoting and financing blood screening and injection safety initiatives to ensure that these cheap and effective interventions are implemented worldwide.

## DIAGNOSTICS FOR HCV: TACKLING THE BOTTLENECK

The advent of highly efficacious direct-acting antiviral (DAA) treatment has revolutionized the therapeutic landscape for chronic HCV infection, but less attention has been paid to screening and diagnosis. Given the nature of the infection, asymptomatic HCV-infected individuals are unlikely to seek health care [41]. Consequently, WHO targets of 90% of active infections diagnosed by 2030 [1] are aspirational, outstripping the diagnosis coverage achieved even in those countries that have been most successful in identifying infected individuals, such as France, Australia, and Sweden [42]. Currently, a 2-step process for diagnosing active HCV infection is usually required: a serological test to screen for exposure, followed by an HCV RNA nucleic acid test (NAT) to confirm viremia [43]. This 2-step process inevitably leads to patient loss to follow-up (LTFU) [44–46]. Furthermore, in LMIC, NAT is economically challenging, and the specialized laboratory staff and equipment are often not available [47, 48].

Alternatively, serum HCV core antigen quantification (HCVcAg) can be used as a surrogate marker for HCV viremia. Testing HCVcAg is a relatively low cost (as low as US$10 per sample [49]), fully automated, and a commercially available assay that can be performed on the Abbott ARCHITECT platform, making it an attractive test for resource-limited settings [49, 50]. Employing HCVcAg testing while still dependent on a centralized testing facility can “uncouple” the sample collection from the testing site through the use of dried blood spot (DBS) samples [51, 52]: While whole blood and plasma samples require prompt transport to the laboratory or refrigeration, DBS samples can be stored at room temperature for several weeks [53]. The robustness of this sample storage technique makes it ideal for decentralizing testing, which is attractive for resource-limited settings [43]. Though testing DBS samples for HCVcAg has been shown to have reduced sensitivity compared with using serum samples [52, 54], the low cost and uncoupling of sample and testing site suggest a role for DBS testing in marginalized populations unlikely to present at centralized testing facilities [54].

The development and validation of RDTs has become a research priority. The WHO has prequalified 2 HCV RDTs: SDBioline (SDBioline, Gyeonggi-do, Republic of Korea) and Oraquick (OraSure Technologies Inc., Bethlehem, PA) [55, 56], an oral fluid-based point-of-care (POC) test with a comparable performance to third-generation enzyme immunoassays (EIAs) [57]. POC testing has been shown to improve HIV linkage to care in LMIC and to be cost-effective [58-60, S61]. Combination RDTs for HIV, HBV, and HCV have also been shown to increase uptake and receipt of results relative to laboratory testing [S62].

Finally, the adoption of pangenotypic DAA regimens may eliminate the requirement for genotype testing entirely [53]. All such developments can simplify and strengthen the diagnosis and treatment cascade, removing potential causes of LTFU, and will be crucial in reaching diagnosis and treatment targets. Active implementation of affordable POC testing at the primary care level will be essential to upscale identification and linkage to care of infected individuals.

## PROVIDING CARE: ACCESSING HARD-TO-REACH HCV-INFECTED POPULATIONS

In most countries, anti-HCV prevalence is well below 10% in the general population [S63] but significantly greater in high-risk populations. The most studied population is PWID, in which anti-HCV prevalence can exceed 90% [S64], but men who have sex with men (MSM) and other recreational drug users are increasingly being recognized as significant at-risk populations [S65, S66]. Such populations are often difficult to reach for a variety of reasons, including stigma and the possibility of prosecution [S67, S68]. Programs specifically designed to address epidemics within these populations are critical to the success of disease burden reduction efforts.

Qualitative [S69, S70] and quantitative [S71–S73] research has shown that PWID are interested in engaging with health care services for HCV testing and treatment; there is clear evidence of successful treatment outcomes in this group [S74], yet treatment in PWID remains suboptimal [S75,S76]. Health care provider concerns can act as barriers to HCV treatment. Such concerns may include the presence of comorbidities, the belief that there will be adherence issues, and difficulties managing side effects [S77]. From a patient perspective, there are several factors that may reduce the likelihood of accessing HCV treatment, including negative experiences within health care systems [S78, S79], low literacy rates [S80, S81], inadequate communication with health care providers regarding the nature of the disease and treatment options [S79], and social factors such as discrimination, the threat of stigmatization, and criminalization [S81].

Reviews of treatment models in high-income countries suggest that effective approaches to improving rates of HCV diagnosis and treatment integrate HCV services into addiction care units [S82]. Focusing on treatment of addiction [S83] and encouraging positive feedback between health care staff and PWID [S84] improve outcomes. Such multidisciplinary approaches reduce noncompliance even among homeless or active illicit drug users [S85]. Similar approaches can be utilized in general practitioner services; a study in the United Kingdom demonstrated how specialist nurses seconded to primary care facilities can offer screening to at-risk individuals, considerably improving HCV detection [S86]. An alternative approach may be treatment as prevention: Modeling suggests that it may be effective at curbing transmission in both PWID [S87] and MSM [S88].

In LMIC, many patients, in addition to those discussed above, are hard to reach through lack of health care services. In such scenarios, HCV screening could be integrated into existing care delivery models for HIV and tuberculosis (TB) [S89, S90]. Success of HIV treatment roll-out offers valuable lessons [43]: Community-based testing improves uptake among high–CD4 count individuals compared with facility-based approaches [S91], and we propose that the impressive improvements in HIV case finding could be replicated for HCV using similar methods.

## TREATING HBV INFECTION: SETTING GLOBAL STANDARDS TO SIMPLIFY CARE

Achieving ambitious WHO testing and treatment targets for HBV requires careful consideration of the challenges of scale-up; discussion should focus on simplified models of care and methods of wide-scale testing and treatment, particularly in LMICs.

Treatment for CHB targets individuals with, or at risk for, advanced liver disease, and aims to suppress virus replication, halt disease progression, prevent complications, and avert HBV-related deaths [S92–S94]. Several antiviral agents approved for the treatment of CHB are available in developed countries, with regional guidelines outlining a continuum of care and recommendations on who to treat, how to treat, and when to stop treatment [S95–S96]. These documents, while based on evidence of drug efficacy and benefits of treatment, are not adapted for use in countries where CHB is endemic and HBV-related mortality is highest [2]. WHO guidelines for the prevention and treatment of CHB attempt to address this unmet need [S94]. Tenofovir is the recommended antiviral in resource-limited settings, with entecavir recommended in children aged 2–11 years. The guidelines encourage the use of clinical parameters (clinical diagnosis of cirrhosis) and/or noninvasive tests (APRI score > 2) to assess severity of liver disease.

WHO guidelines suggest that treatment should be targeted at those with the highest risk of disease progression, based on the detection of persistently raised alanine aminotransferase (ALT), and HBV DNA levels greater than 20 000 IU/mL in those older than age 30 years [S94]. All cirrhotics should be treated regardless of ALT levels, HBeAg status, or HBV DNA levels. The simplicity of administration of these antivirals, their tolerability and safety profiles, and their high barrier to resistance make them ideal for long-term use in regions where close monitoring and management of adverse effects may not be feasible.

While there is consensus that cirrhotics require lifelong treatment, the safety of stopping therapy in noncirrhotic patients remains less clear. Most international guidelines recommend discontinuation of therapy for noncirrhotic HBeAg-positive individuals who show evidence of HBeAg loss and seroconversion to antibody to HBeAg (anti-HBe), undetectable HBV DNA, and who complete at least 12 months of consolidation therapy [S94]. Discontinuation is only recommended when ALT and HBV DNA levels can be monitored, as a high proportion relapse after stopping treatment [S94, S97], placing an additional financial strain on health care systems. Antiviral therapy can be stopped in noncirrhotic HBeAg-negative individuals at least 12 months following loss of HBsAg and achieving anti-HBs status [S98]. This needs to be accompanied by sustained virological suppression and normalization of ALT, with off-treatment monitoring for relapse. With only a small proportion of patients achieving HBsAg loss, and with the need for regular monitoring for reactivation and flares after cessation of antiviral therapy in both HBeAg-positive and -negative individuals, we believe it is advisable at present to treat all patients indefinitely [S98]. The additional financial burden that such lifelong treatment imposes, however, as well as challenges to maintaining people in the cascade of care, must be acknowledged when adopting this approach.

An additional complication is access to treatment in LMICs: despite generic tenofovir costing less than US$50 per annum and being accessible to HIV-HBV co-infected individuals as part of antiretroviral therapy, many HBV-monoinfected individuals cannot access antiviral therapy or have to pay out of pocket. National health care systems need to ensure funded, sustainable access to antiviral therapy for HBV-mono-infected individuals, implemented in concert with diagnosis scale-up strategies.

Treatment programs need to be accompanied by wide-scale HBV testing, particularly in LMICs, where screening rates are low. Community-based testing and treatment for chronic HBV infection has been shown to be feasible: In The Gambia, the Prevention of Liver Fibrosis and Cancer in Africa (PROLIFICA) study showed that POC HBV tests perform well in African community settings [S99], and subsequently demonstrated that community-based screening could achieve high coverage and good linkage to care [S100] while remaining cost-effective [S101].

## THE COST OF DIRECT-ACTING ANTIVIRALS FOR HCV: HOW PROGRESS CAN BE MADE

A 12-week DAA course costs in excess of US$70 000 in the United States today [S102]. With an estimated 71 million active HCV infections globally [1], such prices render any program aimed at global HCV elimination unrealistic. In 2016, the WHO reported a range of approaches adopted by several countries to demonstrate how barriers to treatment can be overcome [S103].

In LMICs where patents have not been filed or remain under examination, a market for low-cost, generic versions of patented drugs can be created. Sofosbuvir is not patented in Egypt, and a 28-day supply currently costs under US$30 [S104]. India has used this approach to facilitate production of generics, but a recent decision to grant a patent to Gilead for Sofosbuvir may damage India’s role as an HCV generics producer [S105]. Pharmaceutical companies have, in places, awarded voluntary license agreements to permit local companies to produce generics. As of August 2015, Gilead had 11 such agreements with Indian companies [S106]. Additionally, the originator company of Daclatasvir has signed an agreement with the Medicines Patent Pool to enable sublicensing to multiple generic manufacturers in 112 LMICs [S103]. Many middle-income countries are viewed as having market potential and are excluded from these agreements, including China, Brazil, and Thailand [S106]. More generally, complexities of voluntary licensing fragment the market, ensuring that pricing power remains in the hands of pharmaceutical companies [S107]. This problem can be circumvented by development of new therapies: The Drugs for Neglected Diseases Initiative (DNDi) has obtained the license for a new HCV DAA, ravidasvir. This has allowed it to begin production of the drug through an Egyptian firm, Pharco Pharmaceuticals, without the need to maximize profit [S108].

High-income countries can bulk purchase to reduce costs; no country has attempted this more ambitiously than Australia. The government agreed to a AU$1 billion (US$0.73 billion) program to treat 62 000 individuals, corresponding to per-treatment costs of around US$12 000 [S109]. The true novelty of the Australian approach is that if expenditure exceeds up-front cost, the price of drugs decreases, potentially to 0 [S109]. Australia has, in effect, created a subscription system, paying a fixed amount and treating as many Australians as it can.

What ultimately unites these approaches is their customized nature; some countries have had success in reducing prices, but others risk being left behind. A unified approach must be taken to ensure that high-quality, low-cost drugs are available regardless of location. Pooled procurement is one method for achieving this, an approach pioneered by the Global Fund to tackle the HIV, malaria, and TB epidemics [S110], and attempts are underway to apply this approach to viral hepatitis [S111]. Such schemes could be transformative; however, they will rely on capital and political will to achieve the scale required for the program to be a success.

### Egypt as a Case Study

Egypt serves as a model for HCV diagnosis and treatment scale-up [S104, S112]. This country, with the world’s highest HCV prevalence, has increased HCV treatment numbers to the hundreds of thousands [S103] and intends to treat 5 million patients by 2030 [S104]. It has achieved this by slashing HCV treatment costs and empowering dozens of diagnostic facilities. Furthermore, by simplifying the diagnostic process through implementing the first universal test and treat strategy, it has reduced the time from initial diagnosis to treatment to as little as one week in some centers [104]. Egypt offers an invaluable example to policy makers, not only as an exemplar of how viral hepatitis interventions can be scaled-up quickly and intensively, but also as a guide for how to tackle the problems that may be faced with such ambitious interventions.

### Recommendations

Egypt demonstrates what can be accomplished with regards to HCV diagnosis and treatment, given sufficient political will, state coordination, and investment. Across the world, efforts to address viral hepatitis, both HBV and HCV, should focus on providing universal HBV birth-dose vaccination within 24 hours of birth, establishing hemovigilance schemes to prevent transmission of blood-borne viruses, providing nonreusable syringes and educating health staff in injection safety, adopting new diagnostic technologies to expand access to screening, specializing intervention efforts for vulnerable high-risk groups to reduce transmission where it is highest, treating all HBV-infected patients indefinitely to minimize potential harm caused by discontinuation, and collaborating across the continent to drive down HCV drug costs to ensure that, once treatment has been accessed, it is affordable.

## Supplementary Data

Supplementary materials are available at *Open Forum Infectious Diseases* online. Consisting of data provided by the authors to benefit the reader, the posted materials are not copyedited and are the sole responsibility of the authors, so questions or comments should be addressed to the corresponding author.

ofx252_suppl_supplementary_appendix
