# Supplementary material for: Aiming at the Global Elimination of Viral Hepatitis: Challenges Along the Care Continuum
Source: Open Forum Infect Dis. 2017 Nov 17;5(1):ofx252. doi: 10.1093/ofid/ofx252 (PMC5767952; doi:10.1093/ofid/ofx252)
Supplement: ofx252_suppl_supplementary_appendix [file ofx252_suppl_supplementary_appendix.docx]

Supplementary Appendix: Aiming at the Global Elimination of Viral Hepatitis: Challenges along the Care Continuum

61. Hyle EP, Jani IV, Lehe J, et al. The clinical and economic impact of point-of-care CD4 testing in Mozambique and other resource-limited settings: a cost-effectiveness analysis. PLoS Med **2014**; 11:e1001725.

62. Bottero J, Boyd A, Gozlan J, et al. Simultaneous human immunodeficiency virus-hepatitis B-hepatitis C point-of-care tests Improve outcomes in linkage-to-care: results of a randomized control trial in persons without healthcare coverage. Open Forum Infect. Dis. **2015**; 2:ofv162.

63. The Polaris Observatory HCV Collaborators. Global prevalence and genotype distribution of hepatitis C virus infection in 2015: a modelling study. Lancet Gastroenterol. Hepatol. **2017**; 2:161–176.

64. Aceijas C, Rhodes T. Global estimates of prevalence of HCV infection among injecting drug users. Int. J. Drug Policy **2007**; 18:352–358.

65. Jordan AE, Perlman DC, Neurer J, Smith DJ, Des Jarlais DC, Hagan H. Prevalence of hepatitis C virus infection among HIV+ men who have sex with men: a systematic review and meta-analysis. Int. J. STD AIDS **2017**; 28:145–159.

66. Xu H, Yu G, Sun H, et al. Use of parenteral caffeinum natrio-benzoicum: an underestimated risk factor for HCV transmission in China. BMC Public Health **2015**; 15. Available at: http://www.biomedcentral.com/1471-2458/15/928. Accessed 26 April 2016.

67. Tkatchenko-Schmidt E, Renton A, Gevorgyan R, Davydenko L, Atun R. Prevention of HIV/AIDS among injecting drug users in Russia: Opportunities and barriers to scaling-up of harm reduction programmes. Health Policy **2008**; 85:162–171.

68. Mimiaga MJ, Safren SA, Dvoryak S, Reisner SL, Needle R, Woody G. ‘We fear the police, and the police fear us’: structural and individual barriers and facilitators to HIV medication adherence among injection drug users in Kiev, Ukraine. AIDS Care **2010**; 22:1305–1313.

69. Doab A, Treloar C, Dore GJ. Knowledge and attitudes about treatment for hepatitis C virus infection and barriers to treatment among current injection drug users in Australia. Clin. Infect. Dis. **2005**; 40:S313–S320.

70. Treloar C, Holt M. Drug treatment clients’ readiness for hepatitis C treatment: implications for expanding treatment services in drug and alcohol settings. Aust. Health Rev. **2008**; 32:570–576.

71. Canfield KM, Smyth E, Batki SL. Methadone maintenance patients’ knowledge, attitudes, beliefs, and experiences concerning treatment for hepatitis C virus infection. Subst. Use Misuse **2010**; 45:496–514.

72. Grebely J, Genoway KA, Raffa JD, et al. Barriers associated with the treatment of hepatitis C virus infection among illicit drug users. Drug Alcohol Depend. **2008**; 93:141–147.

73. Strathdee SA, Latka M, Campbell J, et al. Factors associated with interest in initiating treatment for hepatitis C Virus (HCV) infection among young HCV-infected injection drug users. Clin. Infect. Dis. **2005**; 40. Available at: http://escholarship.org/uc/item/0g44g60r.pdf%3Faction=transientDownload. Accessed 21 February 2017.

74. Hellard M, Sacks‐Davis R, Gold J. Hepatitis C treatment for injection drug users: a review of the available evidence. Clin. Infect. Dis. **2009**; 49:561–573.

75. Mehta SH, Genberg BL, Astemborski J, et al. Limited uptake of hepatitis C treatment among injection drug users. J. Community Health **2008**; 33:126–133.

76. Lindenburg CEA, Lambers FAE, Urbanus AT, et al. Hepatitis C testing and treatment among active drug users in Amsterdam: results from the DUTCH-C project. Eur. J. Gastroenterol. Hepatol. **2011**; 23:23–31.

77. Edlin BR, Kresina TF, Raymond DB, et al. Overcoming barriers to prevention, care, and treatment of hepatitis C in illicit drug users. Clin. Infect. Dis. **2005**; 40:S276–S285.

78. Swan D, Long J, Carr O, et al. Barriers to and facilitators of hepatitis C testing, management, and treatment among current and former injecting drug users: a qualitative exploration. AIDS Patient Care STDs **2010**; 24:753–762.

79. Schackman BR, Teixeira PA, Beeder AB. Offers of hepatitis C care do not lead to treatment. J. Urban Health **2007**; 84:455–458.

80. Treloar C, Hull P, Bryant J, Hopwood M, Grebely J, Lavis Y. Factors associated with hepatitis C knowledge among a sample of treatment naive people who inject drugs. Drug Alcohol Depend. **2011**; 116:52–56.

81. Harris M, Rhodes T. Hepatitis C treatment access and uptake for people who inject drugs: a review mapping the role of social factors. Harm. Reduct. J. **2013**; 10:7.

82. Bruggmann P, Litwin AH. Models of care for the management of hepatitis C virus among people who inject drugs: one size does not fit all. Clin. Infect. Dis. **2013**; 57:S56–S61.

83. Dimova RB, Zeremski M, Jacobson IM, Hagan H, Des Jarlais DC, Talal AH. Determinants of hepatitis C virus treatment completion and efficacy in drug users assessed by meta-analysis. Clin. Infect. Dis. **2013**; 56:806–816.

84. Rich ZC, Chu C, Mao J, et al. Facilitators of HCV treatment adherence among people who inject drugs: a systematic qualitative review and implications for scale up of direct acting antivirals. BMC Public Health **2016**; 16. Available at: http://bmcpublichealth.biomedcentral.com/articles/10.1186/s12889-016-3671-z. Accessed 21 February 2017.

85. Wilkinson M, Crawford V, Tippet A, et al. Community-based treatment for chronic hepatitis C in drug users: high rates of compliance with therapy despite ongoing drug use. Aliment. Pharmacol. Ther. **2009**; 29:29–37.

86. Jack K, Willott S, Manners J, Varnam MA, Thomson BJ. Clinical trial: a primary-care-based model for the delivery of anti-viral treatment to injecting drug users infected with hepatitis C. Aliment. Pharmacol. Ther. **2009**; 29:38–45.

87. Martin NK, Vickerman P, Grebely J, et al. Hepatitis C virus treatment for prevention among people who inject drugs: modeling treatment scale-up in the age of direct-acting antivirals. Hepatology **2013**; 58:1598–1609.

88. Martin NK, Thornton A, Hickman M, et al. Can Hepatitis C Virus (HCV) Direct-Acting Antiviral Treatment as Prevention Reverse the HCV Epidemic Among Men Who Have Sex With Men in the United Kingdom? Epidemiological and Modeling Insights. Clin. Infect. Dis. **2016**; 62:1072–1080.

89. Suthar AB, Harries AD. A public health approach to hepatitis C control in low- and middle-income countries. PLOS Med. **2015**; 12:e1001795.

90. Kim JY, Farmer P, Porter ME. Redefining global health-care delivery. Lancet **2013**; 382:1060–1069.

91. Suthar AB, Ford N, Bachanas PJ, et al. Towards universal voluntary HIV testing and counselling: a systematic review and meta-analysis of community-based approaches. PLoS Med. **2013**; 10:e1001496.

92. Terrault NA, Bzowej NH, Chang K-M, Hwang JP, Jonas MM, Murad MH. AASLD guidelines for treatment of chronic hepatitis B. Hepatology **2016**; 63:261–283.

93. European Association For The Study Of The Liver. EASL clinical practice guidelines: management of chronic hepatitis B virus infection. J. Hepatol. **2012**; 57:167–185.

94. World Health Organization. Guidelines for the prevention, care and treatment of persons with chronic hepatitis B infection. World Health Organization, 2015. Available at: http://apps.who.int/iris/bitstream/10665/154590/1/9789241549059_eng.pdf. Accessed 10 July 2017.

95. Sarin S, Kumar M, Lau G, et al. Asian-Pacific clinical practice guidelines on the management of hepatitis B: a 2015 update. Hepatol. Int. **2016**; 10:1–98.

96. Bottero J, Brouard C, Roudot-Thoraval F, et al. 2014 French guidelines for hepatitis B and C screening: a combined targeted and mass testing strategy of chronic viruses namely HBV, HCV and HIV. Liver Int. **2016**; 36:1442–1449.

97. Jeng W-J, Sheen I-S, Chen Y-C, et al. Off-therapy durability of response to entecavir therapy in hepatitis B e antigen-negative chronic hepatitis B patients. Hepatology **2013**; 58:1888–1896.

98. Chong CH, Lim SG. When can we stop nucleoside analogues in patients with chronic hepatitis B? Liver Int. **2017**; 37:52–58.

99. Njai HF, Shimakawa Y, Sanneh B, et al. Validation of rapid point-of-care (POC) tests for detection of hepatitis B surface antigen in field and laboratory settings in the Gambia, Western Africa. J. Clin. Microbiol. **2015**; 53:1156–1163.

100. Lemoine M, Shimakawa Y, Njie R, et al. Acceptability and feasibility of a screen-and-treat programme for hepatitis B virus infection in The Gambia: the Prevention of Liver Fibrosis and Cancer in Africa (PROLIFICA) study. Lancet Glob. Health **2016**; 4:e559–e567.

101. Nayagam S, Conteh L, Sicuri E, et al. Cost-effectiveness of community-based screening and treatment for chronic hepatitis B in The Gambia: an economic modelling analysis. Lancet Glob. Health **2016**; 4:e568–e578.

102. GoodRx. Epclusa Prices. 2016. Available at: http://www.goodrx.com/epclusa. Accessed 12 November 2016.

103. World Health Organization. Global report on access to hepatitis C treatment. Focus on overcoming barriers. 2016. Available at: http://apps.who.int/iris/handle/10665/250625. Accessed 10 February 2017.

104. Gomaa A, Allam N, Elsharkawy A, El Kassas M, Waked I. Hepatitis C infection in Egypt: prevalence, impact and management strategies. Hepatic Med. Evid. Res. **2017**; Volume 9:17–25.

105. Ellen F M ’t Hoen. Indian hepatitis C drug patent decision shakes public health community. Lancet **2016**; 387:2272–2273.

106. MSF. Strategies to secure access to generic hepatitis C medicines: overcoming patent and regulatory barriers to secure access to generic hepatitis C medicines. 2015. Available at: https://www.msfaccess.org/sites/default/files/MSF_assets/HepC/Docs/HepC_brief_OvercomingbarriersToAccess_ENG_2015.pdf. Accessed 10 July 2017.

107. Baker BK. Gilead’s proposed hepatitis C medicines license – how badly will it miss the target? 2014. Available at: http://infojustice.org/wp-content/uploads/2014/09/baker09122014.pdf. Accessed 20 November 2016.

108. Drugs for Neglected Diseases initiative (DNDi). Drugs for Neglected Diseases initiative and Pharco Pharmaceuticals to test affordable hepatitis C regimen with support of Malaysian and Thai governments. 2016. Available at: http://www.dndi.org/2016/media-centre/press-releases/dndi-pharco-hepc-malaysia-thailand/. Accessed 20 November 2016.

109. Alcorn K. Australia shows an alternative to rationing hepatitis C treatment. 2016. Available at: http://www.aidsmap.com/page/3061523/. Accessed 20 November 2016.

110. The Global Fund. Global Fund strategy 2017-2022. 2017. Available at: https://www.theglobalfund.org/media/2531/core_globalfundstrategy2017-2022_strategy_en.pdf. Accessed 31 May 2017.

111. Global Procurement Fund. The Global Procurement Fund. 2017. Available at: http://gprofund.org/access_countries.htm. Accessed 31 May 2017.

112. Elgharably A, Gomaa AI, Crossey MME, Norsworthy PJ, Waked I, Taylor-Robinson SD. Hepatitis C in Egypt - past, present, and future. Int. J. Gen. Med. **2016**; 10:1–6.
